# Estimating sensitivity of seabed habitats to disturbance by bottom trawling based on the longevity of benthic fauna

**DOI:** 10.1002/eap.1731

**Published:** 2018-05-24

**Authors:** Adriaan D. Rijnsdorp, Stefan G. Bolam, Clement Garcia, Jan Geert Hiddink, Niels T. Hintzen, P. Daniel van Denderen, Tobias van Kooten

**Affiliations:** ^1^ Wageningen Marine Research P.O. Box 68 1970 AB IJmuiden The Netherlands; ^2^ Aquaculture and Fisheries Group Wageningen University P.O. Box 338 6700 AH Wageningen The Netherlands; ^3^ The Centre for Environment, Fisheries and Aquaculture Science Pakefield Road Suffolk NR33 0HT United Kingdom; ^4^ School of Ocean Sciences Bangor University Menai Bridge Anglesey LL59 5AB United Kingdom; ^5^ Centre for Ocean Life National Institute of Aquatic Resources Technical University of Denmark Kemitorvet, B‐202 2800 Kongens Lyngby Denmark; ^6^ Institute for Biodiversity and Ecosystem Dynamics University of Amsterdam P.O. Box 94248 1090 GE Amsterdam The Netherlands

**Keywords:** benthic fauna, ecosystem‐based management, effects of trawling, impact assessment, indicators, sea floor

## Abstract

Bottom fishing such as trawling and dredging may pose serious risks to the seabed and benthic habitats, calling for a quantitative assessment method to evaluate the impact and guide management to develop mitigation measures. We provide a method to estimate the sensitivity of benthic habitats based on the longevity composition of the invertebrate community. We hypothesize that long‐lived species are more sensitive to trawling mortality due to their lower pace of life (i.e., slower growth, late maturation). We analyze data from box‐core and grab samples taken from 401 stations in the English Channel and southern North Sea to estimate the habitat‐specific longevity composition of the benthic invertebrate community and of specific functional groups (i.e., suspension feeders and bioturbators), and examine how bottom trawling affects the longevity biomass composition. The longevity biomass composition differed between habitats governed by differences in sediment composition (gravel and mud content) and tidal bed‐shear stress. The biomass proportion of long‐lived species increased with gravel content and decreased with mud content and shear stress. Bioturbators had a higher median longevity than suspension feeders. Trawling, in particular by gears that penetrate the seabed >2 cm, shifted the community toward shorter‐lived species. Changes from bottom trawling were highest in habitats with many long‐lived species (hence increasing with gravel content, decreasing with mud content). Benthic communities in high shear stress habitats were less affected by bottom trawling. Using these relationships, we predicted the sensitivity of the benthic community from bottom trawling impact at large spatial scale (the North Sea). We derived different benthic sensitivity metrics that provide a basis to estimate indicators of trawling impact on a continuous scale for the total community and specific functional groups. In combination with high resolution data of trawling pressure, our approach can be used to monitor and assess trawling impact and seabed status at the scale of the region or broadscale habitat and to compare the environmental impact of bottom‐contacting fishing gears across fisheries.

## Introduction

Human activities are putting pressure on ecosystems, potentially jeopardizing their structure and functioning. Sustainable ecosystem management requires information on the extent and intensity of human activities, as well as on the sensitivity of ecosystems to these activities (Sanderson et al. 2002, Halpern et al. 2008). The sensitivity of an ecosystem to human pressures depends on the direct effect of these pressures on the ecological system (resistance) and the capacity of a system to recover (resilience; Vieira et al. 2004, Nimmo et al. 2015). The study of biological traits has been promoted as a powerful tool to understand ecological impacts from human pressures (Bremner 2008, Haddad et al. 2008) and has been used to develop indicators of impact and status for managing human activities (Menezes et al. 2010, Beauchard et al. 2017).

Here we use a biological trait approach to assess the sensitivity of seabed habitats to the effect of bottom trawl fishing disturbance (i.e., demersal trawls and shellfish dredges). Bottom trawling occurs over large parts of the continental shelf and is a dominant human pressure in these habitats, whereas mining, dredging, and sand and gravel extraction are more localized activities (Eastwood et al. 2007, Foden et al. 2011). The footprint of bottom trawling on the European continental shelf varies between 53% and 99% by habitat type (Eigaard et al. 2017). Bottom trawls physically disturb seabed sediments, damage biogenic structures and kill benthic invertebrates, affecting both structure and function of the benthic ecosystem (Dayton et al. 1995, Kaiser 1998, Thrush and Dayton 2002). Bottom trawling shows a heterogeneous distribution with some areas trawled several times per year and other areas less frequently or not at all (Rijnsdorp et al. 1998, Lee et al. 2010, Gerritsen et al. 2013, van Denderen et al. 2015b). The impact of trawling differs between fishing gears, and is largely determined by the penetration depth of the gear and frequency of trawl passes (Eigaard et al. 2016, Hiddink et al. 2017). The impact further depends on the resistance of the benthic organisms, determined by their position in the sediment, their fragility, and their size (Bergman and van Santbrink 2000, Bolam et al. 2014). Robust taxa, e.g., those protected by a hard shell, are likely to suffer a lower mortality rate when exposed to a trawl gear compared to more fragile taxa such as worms (Collie et al. 2000, Kaiser et al. 2006). In general, we expect that the resilience, e.g., the recovery rate after a disturbance event, depends on life history characteristics such as age at first maturation and mode of reproduction. Opportunistic species (*r* strategist) are able to recover fast as they mature early and produce many offspring. At the other extreme, *K* strategists mature at a late age and produce fewer offspring, which lead to slower recovery potentials (MacArthur and Wilson 1967, Hoenig 1983, Charnov 1993, van Savage et al. 2004).

In order to assess the impact of bottom trawling on the seabed at regional scales, as required in the European Union under the Marine Strategy Framework Directive (EC 2008), there is a need for a quantitative assessment methodology to estimate differences in habitat sensitivity across a wide range of seabed habitats at large spatial scales (Rice et al. 2012, ICES, 2017a; Kenny et al. 2018). Methods based on the longevity composition of benthic ecosystems may be a good starting point (Thrush et al. 2005, Rijnsdorp et al. 2016). Longevity is related to body size and reproductive traits and will affect the resistance and resilience of species (Hoenig 1983, Roff 1992, Charnov 1993). It is well known that trawling shifts the community composition of benthos toward short‐lived species (Tillin et al. 2006, de Juan et al. 2007, van Denderen et al. 2015a).

In this paper, we examine how the longevity composition of benthic communities varies across benthic habitats in relation to natural disturbance, sediment composition, and trawling intensity. The sensitivity of the seabed to bottom trawling is estimated from the habitat‐specific longevity composition and by the habitat‐specific effect of bottom trawling on the community composition. The analysis is conducted for the total benthic macroinvertebrate community, as well as for different subsets of taxa representing different ecosystem functions (bioturbators, suspension feeders), and compared between fauna living on the surface and shallow‐ and deep‐burrowing infauna. Habitat sensitivity maps are produced of the critical trawling intensities that reduce the biomass of long‐lived taxa to a certain level. The utility of the longevity composition to estimate indicators for impact, status, and recovery is discussed.

## Material and Methods

### Benthic samples

**Figure 1:**
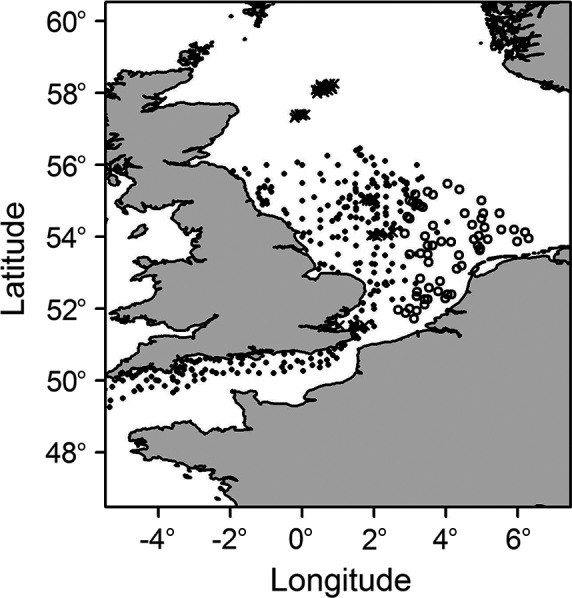
Location of the grab and box‐core sampling stations (solid circles, set 1; open circles, set 2; ×, set 3).

The longevity biomass composition of the benthic community is estimated using benthic samples collected in the North Sea and English Channel with 0.1‐ or 0.078‐m^2^ grabs or box‐cores. These sampling gears provide a quantitative estimate of the biomass of the infaunal and smaller epifaunal part of the macrobenthic community. Data set 1 comprises 299 stations sampled in UK waters between 2000 and 2010 (Bolam et al. 2014). The second and third data sets were compiled by van Denderen et al. (2014, 2015a). Data set 2 comprises 309 samples collected at 62 stations during annual benthic surveys in the Dutch part of the North Sea between 2002 and 2007. Data set 3 comprises 182 samples collected at 40 stations along five trawling gradients across the North Sea in 2002, 2003, or 2004 (Fig. 1). In total, 401 stations were sampled at least once with replicates (between 2 and 6) for 95 stations (Table 1). Replicate samples were collected in the same year (set 3) or sampled over multiple years (set 2). All samples were sieved over a 1‐mm mesh sieve and the retained organisms were identified to the lowest taxonomic level possible. Biomass per taxa was measured as wet mass, except in samples from set 2 where it was measured in ash free dry mass (AFDM). AFDM was converted into wet mass based on conversion factors at the class or phylum level provided by Brey (2008).

**Table 1:** Number of stations and samples by EUNIS‐3 habitat

EUNIS code	Habitat	Number of stations	Number of samples
A5.1	coarse sediment	121	206
A5.2	sandy sediments	230	479
A5.3	muddy sediments	27	82
A5.4	mixed sediments	23	23
Total		401	790

For each sampling station, the sediment characteristics (percent gravel, sand, and mud by mass) were recorded and used to assign a EUNIS habitat for each station (Table 1). The benthos samples represent the range of habitat characteristic of the Greater North Sea study area with a slight under‐representation of muddy and gravely sediments (Appendix S1: Fig. S1).

The trawling intensity for each station is estimated as the average annual swept area ratio (sum of the swept area of the trawling activities over the surface area of the grid cell) of the corresponding 1 × 1 min grid cell for all bottom trawl metiers in the period 2010–2012 (Eigaard et al. 2017). Because the mortality imposed by trawling increases with the penetration depth of the gear (Hiddink et al. 2017), trawling intensity is estimated for the full width of the gears (surface intensity), and for the area swept by the gear components that penetrate >2 cm into the sediment (subsurface intensity). The years for which the trawling intensities are available (2010–2012) do not match the sampling years of the benthos (2000–2010). This will have reduced the statistical power of our analysis but will not have a major effect on the results as the distribution patterns of the fishery at the scale of our study area are rather stable across years (Piet and Quirijns 2009).

### Functional groups

We estimated the longevity biomass composition of the total benthic community, as well as for subsets that represent different sediment positions or specific ecological functions, based on the biological‐trait classification of Bolam et al. (2014). We analysed suspension‐feeding taxa that play an important role in the benthic‐pelagic coupling, bioturbating taxa (diffusive mixers, surface depositors, upward conveyors, downward conveyors) that play an important role in the mixing of the sediments, and groups differing in sediment position of the adults (surface, shallow [0–5 cm], and deep [>5 cm]).

### Habitat variables

**Figure 2:**
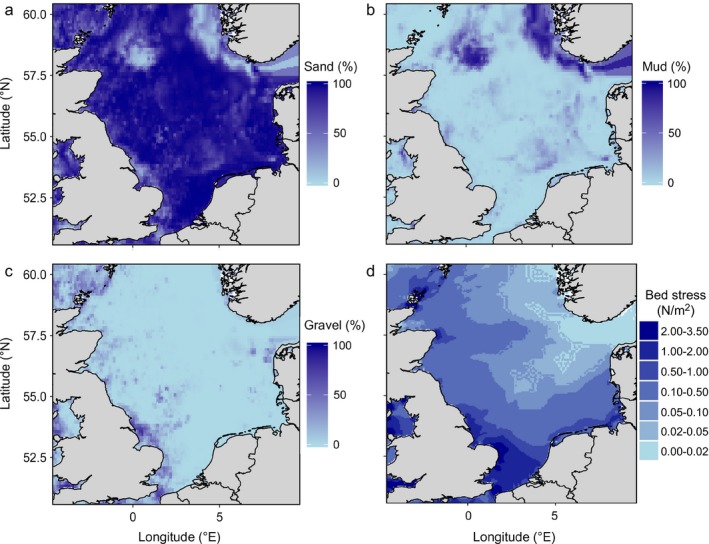
Map of habitat variables: (a) sand, (b) mud, (c) gravel, and (d) tidal bed shear stress (N/m^2^).

Fig. 2 presents the percent sand, mud, and gravel of each 1 × 1 min grid cell obtained from Wilson et al. (2018). Tidal bed shear stress (N/m^2^) was obtained from a hydrodynamic model by John Aldridge (CEFAS) as used in Hiddink et al. (2006) and van Denderen et al. (2015a).

### Longevity composition of the benthic community in different habitats

For each sample, we calculated the cumulative biomass proportion (*B*) by longevity class. Longevity (<1, 1–3, 3–10, >10 yr) information compiled by Bolam et al. (2014) was assigned to each taxon. If the longevity information of a taxon was missing, longevity was assigned based on the information pertaining to a higher taxonomic level.

**Figure 3:**
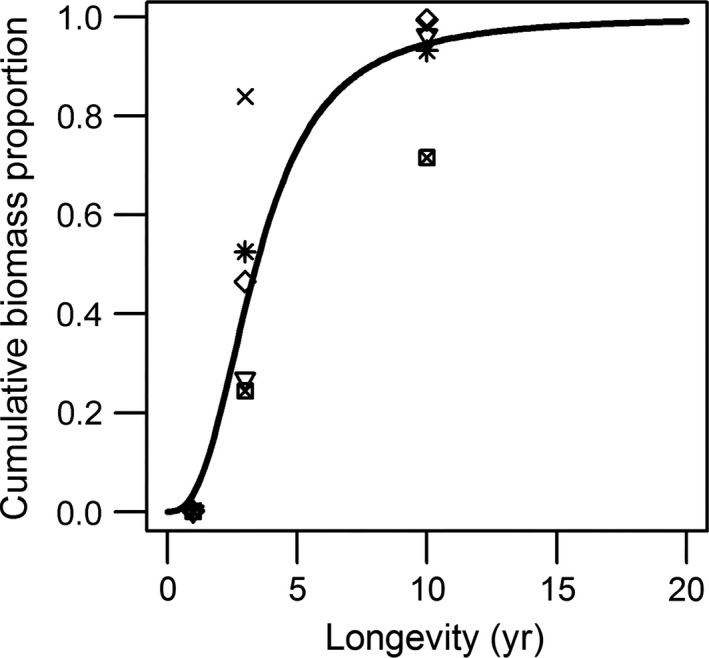
An example of the cumulative biomass–longevity relationship estimated from the observed cumulative biomass by longevity class (1, 1–3, 3–10 yr) in five sampling stations. Different symbols indicate the five different stations.

To convert the longevity classes (*L*_*i*_ = 1, 3, 10) into a continuous scale, we assumed that the biomass proportion in sample *j* with longevity smaller than or equal to *L*_*i*_ (henceforth referred to as the cumulative‐biomass–longevity relationship) is a sigmoidal (logistic) function of *L*_*i*_ (Fig. 3), which starts at 0 and approaches 1 when *L*_*i*_ becomes large (the left side of Eq. (1)). Station and replicates nested within station were included as random effects to take account of the dependency of the cumulative biomass proportions within a sample. The parameter ε represents a binomial error. Random effects were normally distributed with mean 0 and standard deviation σ

(1) $$\ln\frac{B_{\mathrm{ij}}}{1-B_{\mathrm{ij}}}∼\beta_{0}+\beta_{1}\ln\left(L_{i}\right)+\varepsilon_{\mathrm{ij}}$$

Station|replicate∼N(0,σ)

The cumulative biomass—longevity relationship estimated by Eq. (1) is the null model and will be referred to in this paper by the “longevity composition” of the community.

To study the effect of environmental variables on the longevity composition, we added covariables to the null model representing habitat (*H*), trawling intensity (*T*), and the interaction terms between longevity and habitat (*L *× *H*), trawling intensity and habitat (*T *× *H*), and trawling intensity and longevity (*L *× *T*):

(2) $$\begin{array}{cc} \ln\frac{B_{\mathrm{ij}}}{1-B_{\mathrm{ij}}}∼ & \beta_{0}+\beta_{1}\ln\left(L_{i}\right)+\beta_{2}H_{j}+\beta_{3}\ln\left(T_{j}\right)\\ & +\beta_{4}\ln\left(T_{j}\right)\times H_{j}\;+\;\beta_{5}\ln\left(L_{i}\right)\times H_{j}\;\\ & +\;\beta_{6}\ln\left(L_{i}\right)\times \ln\left(T_{j}\right)+\varepsilon_{\mathrm{ij}} \end{array}$$

Station|replicate∼N(0,σ)

The habitat variable *H* represents the continuous habitat covariables (*G*, percent gravel; *M*, percent mud; and *S*, shear stress [N/m^2^]). Sand content was excluded as a covariable because it is highly correlated with both gravel (*r* = 0.70) and mud content (*r* = 0.61). Trawling intensity (*T*) and shear stress were ln(*x* + 0.01)‐transformed to improve the model fit. A small quantity (0.01) was added to each data value to avoid taking the log of zero. We applied the random intercept model because including a random slope on top of the random intercept did not improve the model fit and resulted in poor convergence in some of the more complex models.

The mixed effect model was estimated using the lme4 package (Bates et al. 2015) in R (R Development Core Team 2016). Model selection was based on the Akaike information criterion (AIC).

The median longevity was used to evaluate difference in the longevity composition between habitats, and to study the effect of trawling on the longevity composition. Because we are interested in the uncertainty in the habitat specific biomass–longevity relationships and not in the uncertainty among stations, confidence intervals of the model predictions were based on the fixed effects and their uncertainty.

### Seabed sensitivity

Seabed sensitivity was estimated as the critical trawling intensity (*T*_c_) at which the biomass of long‐lived taxa (>10 yr) is reduced to a proportion (*p*) of the untrawled biomass *B*_ref_

(3) $$\begin{array}{c} T_{c}\;=\;\exp\left[\begin{array}{c} (\ln\left(1-pB_{\mathrm{ref}}/\left(pB_{\mathrm{ref}}\right)\right)-(\beta_{0}\\ +\beta_{1}\ln\left(10\right)\;+\;\beta_{2}H\;+\;\beta_{5}\ln\left(10\right)H)/\\ \left(\beta_{3}\;+\;\beta_{4}H\;+\;\beta_{6}\ln\left(10\right)\right) \end{array}\right] \end{array}$$

The untrawled (reference) biomass of long‐lived taxa *B*_ref_ was calculated from Eq. (2) by setting the longevity at 10 yr and the trawling intensity to a value of 0.01 yr^−1^. The derivation of Eq. (3) is given in Appendix S2.

## Results

### Longevity composition in relation to habitat

**Figure 4:**
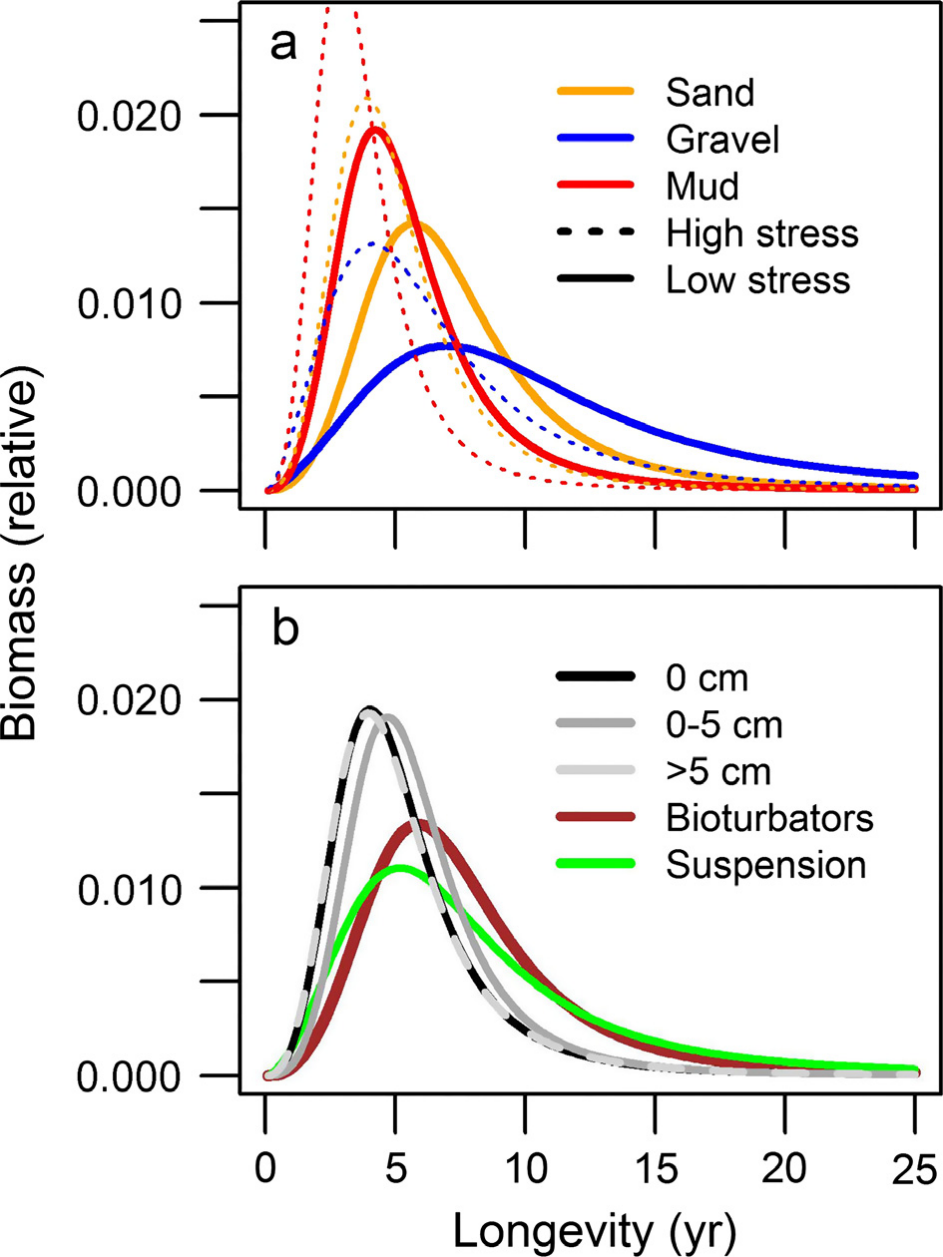
Relative biomass–longevity relationship of the untrawled benthic community for (a) sand (100%), gravel (50% gravel, 50% sand), and mud habitats (50% mud, 50% sand) at high (1 N/m^2^) and low shear stress (0.1 N/m^2^) (model 1); (b) two functional groups (bioturbators, suspension feeders) and three sediment positions (surface 0 cm, shallow 0–5 cm, deep >5 cm) in sandy sediments and low shear stress (models 2–6). The codes in parenthesis refer to the statistical models in Table 2.

**Figure 5:**
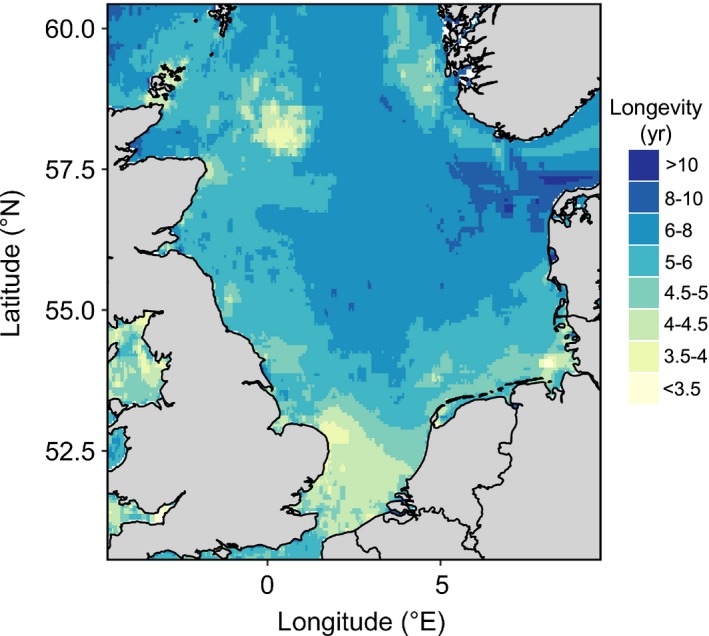
Median longevity of the untrawled habitat estimated by model no. 1.

The longevity composition differs between habitats and is significantly affected by trawling (Table 2). The habitat variables and trawling disturbance explain between 1.8% and 5.0% of the deviance as compared to the null model (Eq. (1)). The low explanatory power of the models reflects the high variability in community composition between individual samples. The most parsimonious model (model 1 in Table 2) includes percent mud, percent gravel, shear stress, and trawling intensity as main effects and significant interactions between longevity and gravel and between shear stress and trawling intensity (Table 3). For untrawled habitats, the biomass proportion of long‐lived species is highest in coarse (gravel) sediments and lowest in muddy sediments. Sandy sediments have an intermediate longevity distribution (Fig. 4a). A higher shear stress shifts the longevity composition toward shorter‐lived taxa. The longevity composition differs between functional groups and is affected by the sediment position (Table 4). Benthos living on the surface and deep taxa consist of relatively more short‐lived taxa as compared to shallow taxa. Suspension feeders have a broader distribution of longevity classes than bioturbators, but their median longevity is lower (Fig. 4b). The map of estimated median longevity reflect the differences in the bed shear stress and sediment composition (Fig. 5).

**Table 2:** Selected mixed‐effect models for the cumulative‐biomass–longevity relationships of all taxa and four different subsets of taxa representing two functional groups (bioturbators, suspension feeders) and different sediment positions (surface, 0–5 cm, >5 cm deep)

Model	Benthos	Model								AIC		Deviance explained (%)	
		*L*	*M*	*G*	*S*	*T*	*T* × *S*	*L* × *G*	*L* × *M*	Sub‐surface trawling intensity	Surface trawling intensity	Sub‐surface trawling intensity	Surface trawling intensity
1	all taxa	x	x	x	x	x	x	x		1,280.6	1,282.7	4.9	4.7
2	bioturbators	x	x	x	x	x	x	x		1,314.1	1,315.5	4.8	4.7
3	suspension feeders	x	x	x	x	x		x		1,618.8	1,617.4	1.8	1.8
4	surface taxa	x	x	x	x	x	x		x	1,436.0	1,431.4	3.7	4.0
5	subsurface taxa (0–5 cm)	x	x	x	x	x	x			1,256.3	1,274.7	5.0	3.6
6	subsurface taxa (>5 cm)	x	x	x				x		1,441.4	1,441.4	2.3	2.3

*Notes*

The Akaike information criterion (AIC) is given for models using the surface and subsurface trawling intensity. The explained deviance is calculated relative to the null model including longevity and the random intercept. *L*, ln(longevity); *M*, %mud; *G*, %gravel; *S*, ln(shear stress); *T*, ln(trawling intensity).

**Table 3:** Parameter estimates of the fixed effects of the continuous habitat model (model 1, Table 2) of the cumulative biomass of all taxa as a function of ln longevity, subsurface trawling intensity (ln[trawling]), %mud (mud), %gravel (gravel), and ln(shear stress)

Variable	Parameter	Estimate	SE	*P*
	intercept	−5.683	0.303	<0.001
*L*	ln(longevity)	3.502	0.165	<0.001
*T*	ln(trawling)	−0.0826	0.0732	0.259
*M*	mud	0.0210	0.0036	<0.001
*G*	gravel	0.0186	0.0114	0.102
*S*	ln(shear stress)	0.0423	0.1288	0.742
*T*: × *S*	ln(trawling) × ln(shear stress)	−0.1182	0.0503	0.019
*L* × *G*	ln(longevity) × gravel	−0.0196	0.0059	0.001

**Table 4:** Test of the difference in the cumulative‐biomass–longevity relationship between suspension feeders and bioturbators and of species that differ in their sediment positions (surface; top 5 cm; >5 cm deep)

Mixed effect model	df	AIC	Deviance	Deviance explained
Functional group (*F*)				
*L* + *M* + *G* + *S* + *T* + *T* × *S* + *L* × *G*	10	2,977.0	2,957.0	
*L* + *M* + *G* + *S* + *T* + *T* × *S* + *L* × *G* + *F* + *F* × *T* + *F* × *S* + *F* × *T* × *S*	14	2,878.6	2,850.6	3.6%
Sediment position (*P*)				
*L* + *M* + *G* + *S* + *T* + *T* × *S*	9	5,374.5	5,356.5	
*L* + *M* + *G* + *S* + *T* + *T* × *S* + *P* + *P* × *T* + *P* × *T* × *S*	17	4,585.2	4,551.2	15.0%

*Note*

*L*, ln(longevity); *M*, %mud; *G*, %gravel; *S*, ln(shear stress); *T*, ln(trawling intensity); *F*, functional group; *P*, sediment position.

### Effect of bottom trawling on the longevity composition

**Figure 6:**
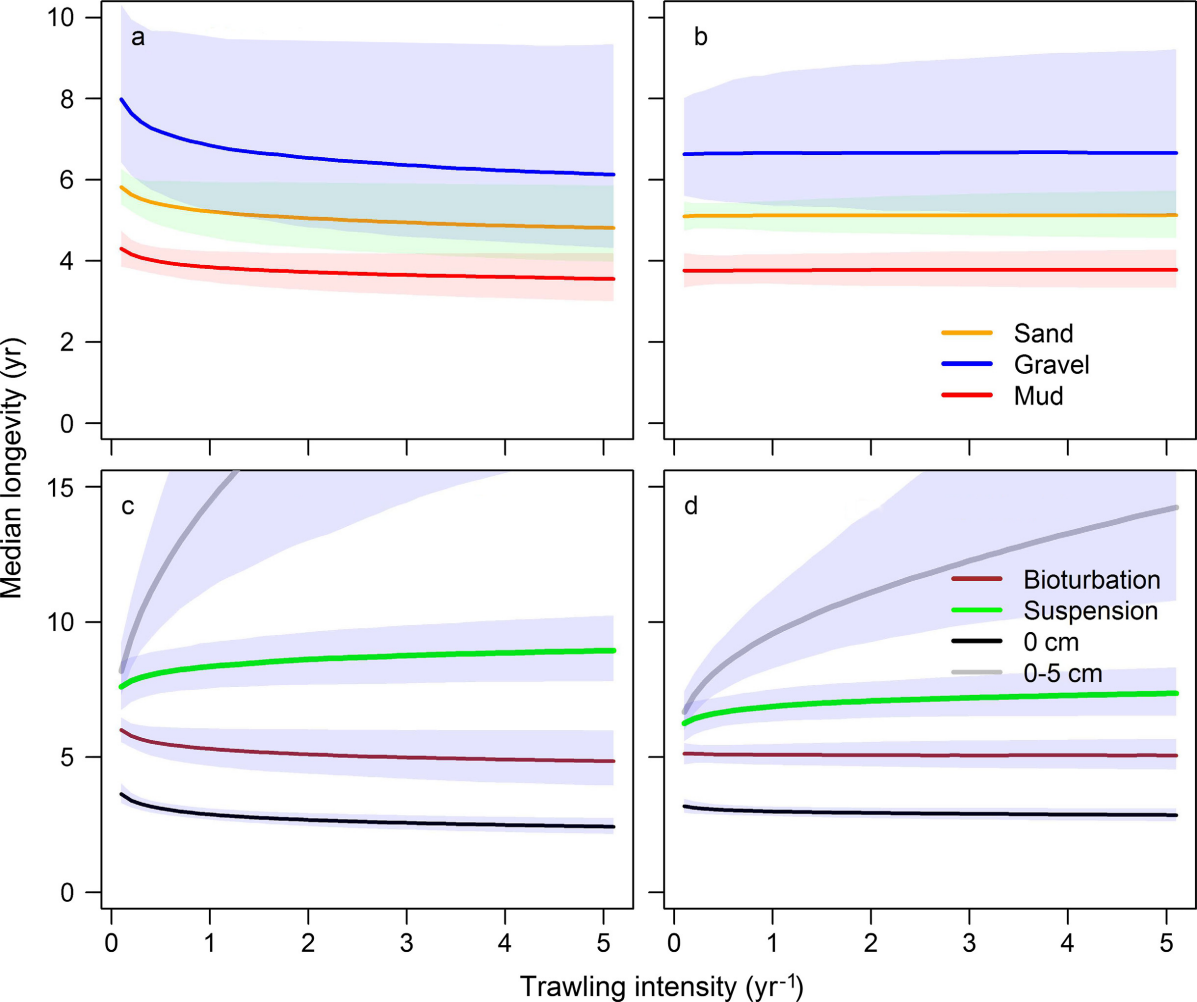
Effect of trawling intensity (subsurface) on the median longevity and 95% confidence limits: all taxa by main habitat types (sand [100% sand], gravel [50% gravel, 50% sand], mud [50% mud, 50% sand]) at (a) low (0.1 N/m^2^) and (b) high (0.5 N/m^2^) shear stress (model 1); subsets representing bioturbating taxa (model 2), suspension feeders (model 3), surface taxa (0 cm, model 4) and shallow taxa (0–5 cm, model no. 5) in a habitat with 100% sand at (c) low (0.1 N/m^2^) and (d) high (0.5 N/m^2^) shear stress. The codes in parenthesis refer to the statistical models in Table 2.

Trawling shifts the longevity composition of the sampled benthos toward shorter living taxa for all taxa and for the subsets of bioturbators and surface taxa (Fig. 6). Trawling does not affect the longevity composition of deep taxa, while the longevity composition of suspension feeders and shallow taxa is shifted to longer lived taxa. Trawling intensity affects the intercept of the longevity composition but not the slope. The models including subsurface trawling intensity show a better fit than the models using the surface trawling intensity, except for suspension feeders and surface taxa (Table 2).

The models show a significant interaction between trawling and tidal‐bed shear stress for all taxa and for the subsets of bioturbators, surface taxa, and shallow taxa, but not for the suspension feeders (Table 2). At low bed stress, the median longevity decreases with increasing trawling intensity for all taxa and for the subsets of bioturbators and surface taxa (Fig. 6a and c). The impact of trawling on median longevity becomes weaker at higher shear stress levels (Fig. 6b and d) and becomes positive above a level of around 0.5 N/m^2^ for all taxa. For the subsets of suspension feeders and shallow taxa the positive effect of trawling intensity on the proportion of long‐lived taxa becomes smaller.

### Habitat sensitivity

**Figure 7:**
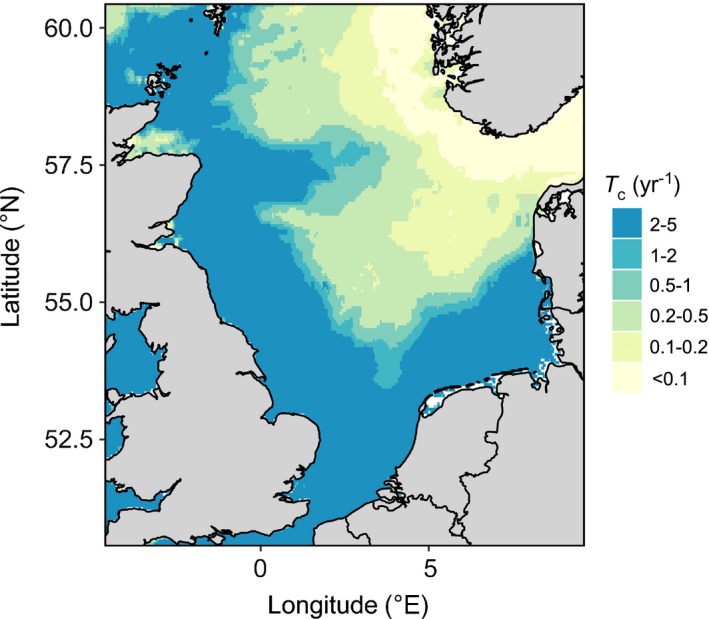
Sensitivity of the seabed to bottom trawling as estimated by the critical trawling intensity (*T*_c_) at which the biomass proportion of long‐lived taxa (longevity ≥ 10 yr) is reduced to 50% of the untrawled reference.

The habitat sensitivity is estimated as the trawling intensity at which the proportion of long‐lived biomass (taxa longevity ≥ 10 yr) is reduced to 50% of the untrawled reference. The analysis shows that in the English Channel, the southern North Sea and along the eastern seaboard of England and Scotland, the benthos is rather insensitive to trawling disturbance (Fig. 7). Trawling intensities of >5 yr^−1^ are required to reduce the proportion of long‐lived taxa to 50%, whereas in the central North Sea trawling intensities <0.2 yr^−1^ already reduce the biomass of long‐lived taxa to 50%.

## Discussion

### Habitat‐specific longevity composition

The longevity composition of the benthic community differed across habitats. Habitats with high gravel content had more long‐lived taxa, while habitats exposed to high shear stress had more short‐lived taxa. The habitat‐specific patterns in the longevity composition observed concur with Southwood (1988), who argued that the habitat provides the templet for organisms to adapt their life history strategy. Being short‐lived is adaptive in environments with a high frequency of disturbance, because it minimizes the chance for an individual to encounter a disturbance during its lifetime and dying before having the chance to reproduce. The life‐history types selected along this disturbance axis match the *r*–*K* selection continuum (MacArthur and Wilson 1967), which has stimulated trait‐based analysis of the community to develop indicators for the state of the ecosystem (Menezes et al. 2010, Beauchard et al. 2017) and to explore consequences for the dynamics of marine populations (Montero‐Serra et al. 2018).

We used the relationships between longevity and the environmental conditions to predict the longevity distribution of the benthic communities throughout the North Sea. We expect that our observed patterns will apply to similar habitats beyond our study area because longevity is a fundamental life history trait, which will be under selection from the environmental conditions characteristic for a particular habitat (Montero‐Serra et al. 2018). Comparison of the range in habitats represented in our benthic data set with the range observed in the North Sea area shows that our data is biased to areas shallower than 150 m (Appendix S1). For sediment characteristics, however, the sampled and observed ranges are quite similar with a slight over‐representation of sandy sediments as compared to gravely or muddy sediments. Further research is needed to test the hypothesis that the relationships between longevity and environmental conditions will also apply for areas beyond the North Sea. In areas where other environmental variables determine the longevity composition, such as the Baltic Sea where salinity and irregular periods of hypoxia have been shown to affect the longevity composition (Törnroos et al. 2015), further studies are required to determine the appropriate longevity compositions.

### Trawling effects on the longevity composition

We found that trawling shifts the benthic community composition (all taxa) toward shorter‐lived taxa. A similar response was observed in subsets of bioturbating taxa and surface taxa. The shift toward shorter‐lived taxa is expected from life history theory as long‐lived taxa will have a slower pace of life and a slower recovery rate after a disturbance event (Hoenig 1983, Charnov 1993). Long lived species may also experience a higher mortality as longevity scales with body size, and body size scales with trawling mortality (Bergman and van Santbrink 2000).

No effect of trawling was observed on the longevity composition in deep (>5 cm) taxa. This is likely since the sediment position of the adults (representing most biomass) is below the penetration depth of the dominant bottom trawl gears (Eigaard et al. 2017, Hiddink et al. 2017). Yet, although not found in our study, animals may still be impacted as trawling may destroy burrows, or animals may be affected when they temporarily occur higher up in the sediment, for instance during their juvenile phase or when they feed, defecate or reproduce (Bergman and van Santbrink 2000). Finally trawling may negatively affect the quality of the habitat for settlement (Piersma et al. 2001).

For shallow taxa and for suspension feeders, trawling unexpectedly shifted the community toward longer lived taxa. This may reflect selection for resistant taxa that are either robust to disturbance events (e.g., *Amphiura*; Sköld et al. 2018), or are able to escape the gear by burying deeper (e.g., *Ensis*) The fast‐burrowing razor clam *Ensis* can reach very high biomasses in some coastal areas of the Netherlands that are regularly trawled, and may have driven this pattern.

For all taxa and the subsets of bioturbators and shallow taxa, best model fits were obtained when including subsurface trawling intensity, which reflects the disturbance by gear components that penetrate more than 2 cm into the sediment (Eigaard et al. 2016). This suggests that for the infauna and smaller epifauna studied the impact of the gear components such as sweeps that only cause surface abrasion may be small in comparison to the impact of heavier gear components that penetrate into the sediment (Eigaard et al. 2016, O'Neill and Ivanović 2016). Indeed a recent meta‐analysis of trawling experiments showed that the mortality rate imposed by trawling is proportional to the penetration depth (Hiddink et al. 2017). For surface taxa and suspension feeders best model fits were obtained when surface trawling intensity was included suggesting that these taxa may also be vulnerable for surface abrasion.

The adverse effect of trawling on the longevity distribution was particularly pronounced in low‐energy habitats, in agreement with previous findings (Bolam et al. 2014, van Denderen et al. 2015a, Neumann et al. 2016). In high‐energy habitats, the model suggests that trawling shifts the longevity composition toward longer‐lived taxa. This may reflect selection for resistant taxa that are either robust to disturbance events, as previously discussed for suspension feeders and shallow taxa, or have a higher recovery rate.

### Habitat sensitivity

We estimated habitat sensitivity as the trawling intensity required to reduce the proportion of long‐lived taxa to a reference level (here 50%). The sensitivity metric, which takes account of the significant interaction between trawling and bed shear stress, shows that in areas exposed to high shear stress the benthos is rather insensitive to trawling. We used tidal bed shear stress as a proxy for natural disturbance of the seabed, ignoring the disturbance by wave action (Aldridge et al. 2015). This implies that in areas where wave‐induced shear stress is important, such as the Dogger Bank in the North Sea, habitat sensitivity is overestimated. Lambert et al. (2017) showed the seabed biota in the shallow waters of the Cardigan Bay, on the west coast of Wales, to be resilient to scallop dredging. This area has a relative low tidal shear stress but is exposed to strong wave disturbance. Differences in erodability of sediments are also not taken into account (Diesing et al. 2013). The relatively well‐sorted sediments of the southern North Sea, for example, are generally far more mobile than the less‐sorted, more gravelly sediments typically found in the English Channel. Incorporating these processes in our statistical model may result in communities in the southern North Sea showing a lower sensitivity to trawling as compared with those in the English Channel.

The estimated habitat sensitivity combines the resistance and the resilience of the benthos, both of which are related to the longevity. Resistance will be negatively related to longevity, because longer‐lived taxa are likely to be larger and are more likely to build biogenic structures, enhancing their mortality rate to trawling (Clark et al. 2016). Resilience is expected to be negatively related to longevity, as long‐lived species start reproducing later in life and have a lower population growth rate (Hoenig 1983, Charnov 1993).

### Longevity composition and ecosystem functioning

Specific groups of benthic organisms are responsible for ecosystem functions. Suspension feeders feed on organic matter produced in the pelagic zone and their activities enhance the transfer of organic matter from the pelagic zone to the benthic ecosystem (Loo and Rosenberg 1989, Norkko et al. 2001). Bioturbators mix the sediment and enhance the irrigation and exchange of nutrients, whereas bioengineers build structures on the surface of the sea floor and burrows or tubes in the sea floor, and this provides a habitat for other taxa (Thrush et al. 1992, Lohrer et al. 2013, Clark et al. 2016).

As benthic functional groups differ in their longevity composition, a comparative analysis of these longevity compositions can be used to determine which ecological functions are most sensitive to trawling disturbance. Our results show that bioturbators have a higher median longevity than suspension feeders suggesting a higher sensitivity for trawling. The statistical analysis of the effect of trawling on the longevity composition indeed shows a shift to shorter‐lived taxa in bioturbators, while a shift to longer‐lived taxa is observed for suspension feeders.

### Uncertainty and potential bias in the reported longevity compositions

The models explain a rather low percentage of the deviance, reflecting large variability in community composition between samples. Although the models have a low predictive power for the community composition at a specific location, the predictive power for a habitat characterized by specific habitat conditions will be higher as we can ignore the variation in the random effect of the stations.

The longevity–habitat relationships are based on data obtained from box‐core and grab samples and therefore reflect the macrobenthos community. These gear types do not effectively sample the larger epifauna and megafauna component of the seabed (Bergman and van Santbrink 2000). Hence, the biomass of deep burrowing species and larger epifaunal species such as sponges, soft corals, sea stars, crabs, and lobsters will be under‐estimated in our data. Few deep burrowing taxa were recorded in our samples taken in muddy sediment, although these habitats are inhabited by deep burying shrimps and echiurans that may occur beyond the sampling depth of the gears used (Nickell et al. 1995, Kinoshita 2002). Since longevity scales with body size, undersampling of the larger benthos will lead to an underestimation of the proportion of long‐lived taxa. The longevity–habitat relationships reported here could be improved by the inclusion of epifaunal data and by broadening the range of habitat variables.

Since intensive trawling has been carried out for more than a century (Engelhard 2008, Kerby et al. 2012), the community composition of the stations that were not trawled during the study period (2010–2012) may still be affected by historic trawling activities. Therefore, we cannot exclude the possibility that vulnerable taxa have already disappeared from the study area, leading to an underestimation of the impact of trawling (Kaiser et al. 2000, Thrush et al. 2008). This may also apply to habitats where we could not detect an effect of trawling on the longevity composition.

### Synthesis and applications

**Figure 8:**
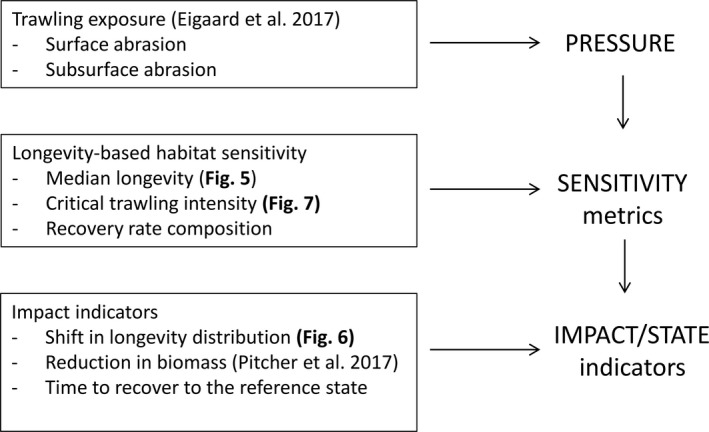
Application of the longevity composition in methods to estimate the effect of bottom trawling on the biodiversity and the benthic biomass.

Our study provides a method to assess the sensitivity of benthic habitats to bottom trawling on a continuous scale. The method allows the derivation of a set of indicators to be used to monitor trawling impact at large, regional scales (North Sea) or between broadscale habitats, and can provide a robust comparison of the impact across different bottom‐contacting fishing gears (Fig. 8). At the core of our approach is the longevity composition of the benthic community, which is estimated from benthic samples, and used to predict the longevity composition of the benthic community typical for a particular habitat. Because of the mechanistic nature of the relationship between habitat variables and life history parameters such as longevity, we expect that the estimated longevity composition can be applied beyond the sampling area. When linked to estimates of the trawling intensity (Eigaard et al. 2017), indicators of trawling impact, such as the relative shift in the longevity composition of the benthic community can be estimated. Since longevity will be related to population growth rate (ICES 2017b), the longevity composition can also be used to estimate the recovery rates of the benthos, which is a requirement of the impact methodology developed by Pitcher et al. (2017) that provides indicators for impact, status and the time required for the benthic biomass recovery (ICES 2017a, b).

Reference values can be set based on biological mechanisms, e.g., the maximum reduction in biomass relative to the carrying capacity at which recruitment may become impaired, or based on an arbitrary choice regarding the acceptable change in indicator value (e.g., maximum allowable recovery time of a habitat). For management application, it is necessary to understand how the indicators vary with trawling disturbance. As shown here, the impact of trawling on benthic habitats is nonlinear. Some habitats are affected much more strongly than other habitats given equal trawling intensities. Furthermore, the relative timing of trawling events is also important, with the first trawl pass typically causing the largest impact (Hiddink et al. 2006). Using relationships between trawling intensity and longevity (this study) and between trawling intensity and mortality and recovery rate (Hiddink et al. 2017, Pitcher et al. 2017), allows for monitoring changes in indicator values over time at local, regional, or broadscale habitat level. The effects of different management scenarios can also be evaluated to guide managers and these indicators can assist in the assessment of trade‐offs between trawling impact and fisheries landings or landing value.

In the interpretation of the metrics and indicators explored in this study, we have assumed that they depend only on the current state of the system (e.g., the trawling distribution). In reality however, the values will change over time even when all else remains equal, as a result of the history of trawling and recovery dynamics of the community. Nevertheless, our analysis provides a basis to monitor the changes in trawling impact or seabed status over time, and can be used to compare the trawling impact of different fisheries taking account of the spatial distribution of trawling in relation to habitat sensitivity (Eigaard et al. 2017), and differences in mortality rate between gears (Hiddink et al. 2017).

Application of our methodology to other areas does not necessarily require intensive sampling of the benthos as long as it is within the range of habitat variables sampled and within habitats where the longevity composition can be expected to be determined by the habitat variables included in the model. To apply the model to a habitat that is not covered by the benthic sampling, or in an area where other variables are expected to affect the longevity composition (e.g., the shelf break or in the Baltic Sea), further studies of the longevity composition are required.

This work shows that the distribution of life‐history characteristics in communities varies with environmental conditions, and that communities in high natural‐disturbance environments with shorter‐lived fauna are less sensitive to anthropogenic disturbance. These insights are widely applicable, and can be used in the spatial management of human activities in all ecosystems by directing human activities toward habitats with short‐lived biota and away from naturally stable habitats with a greater proportion of long‐lived biota.

## Data Availability

Data available from the Dryad Digital Repository: https://doi.org/10.5061/dryad.th2c5f7
