# Supplementary material for: Estimating sensitivity of seabed habitats to disturbance by bottom trawling based on the longevity of benthic fauna
Source: Ecol Appl. 2018 May 24;28(5):1302–12. doi: 10.1002/eap.1731 (PMC6055734; doi:10.1002/eap.1731)
Supplement: Supplementary file 1 [file EAP-28-1302-s001.pdf]

Appendix S1

Ecological Applications

Estimating sensitivity of seabed habitats to disturbance by bottom trawling based on the longevity of benthic fauna.

Adriaan D. Rijnsdorp[1,2], Stefan G. Bolam[3], Clement Garcia[3], Jan Geert Hiddink[4], Niels T. Hintzen[1], P. Daniel van Denderen[5], Tobias van Kooten[1,6]

[1] Wageningen Marine Research, P.O. Box 68, 1970 AB IJmuiden, The Netherlands;

[2] Aquaculture and Fisheries Group, Wageningen University, P.O. Box 338, 6700 AH Wageningen, The Netherlands;

[3] The Centre for Environment, Fisheries and Aquaculture Science, Pakefield Road, Suffolk NR33 0HT, UK;

[4] School of Ocean Sciences, Bangor University, Menai Bridge, Anglesey, LL59 5AB, UK ;

[5] Centre for Ocean Life, National Institute of Aquatic Resources, Technical University of Denmark, Kemitorvet B-202, 2800 Kongens Lyngby, Denmark;

[6] Institute for Biodiversity and Ecosystem Dynamics, University of Amsterdam, Amsterdam, The Netherlands.

Corresponding author: Adriaan D. Rijnsdorp (adriaan.rijnsdorp@wur.nl)

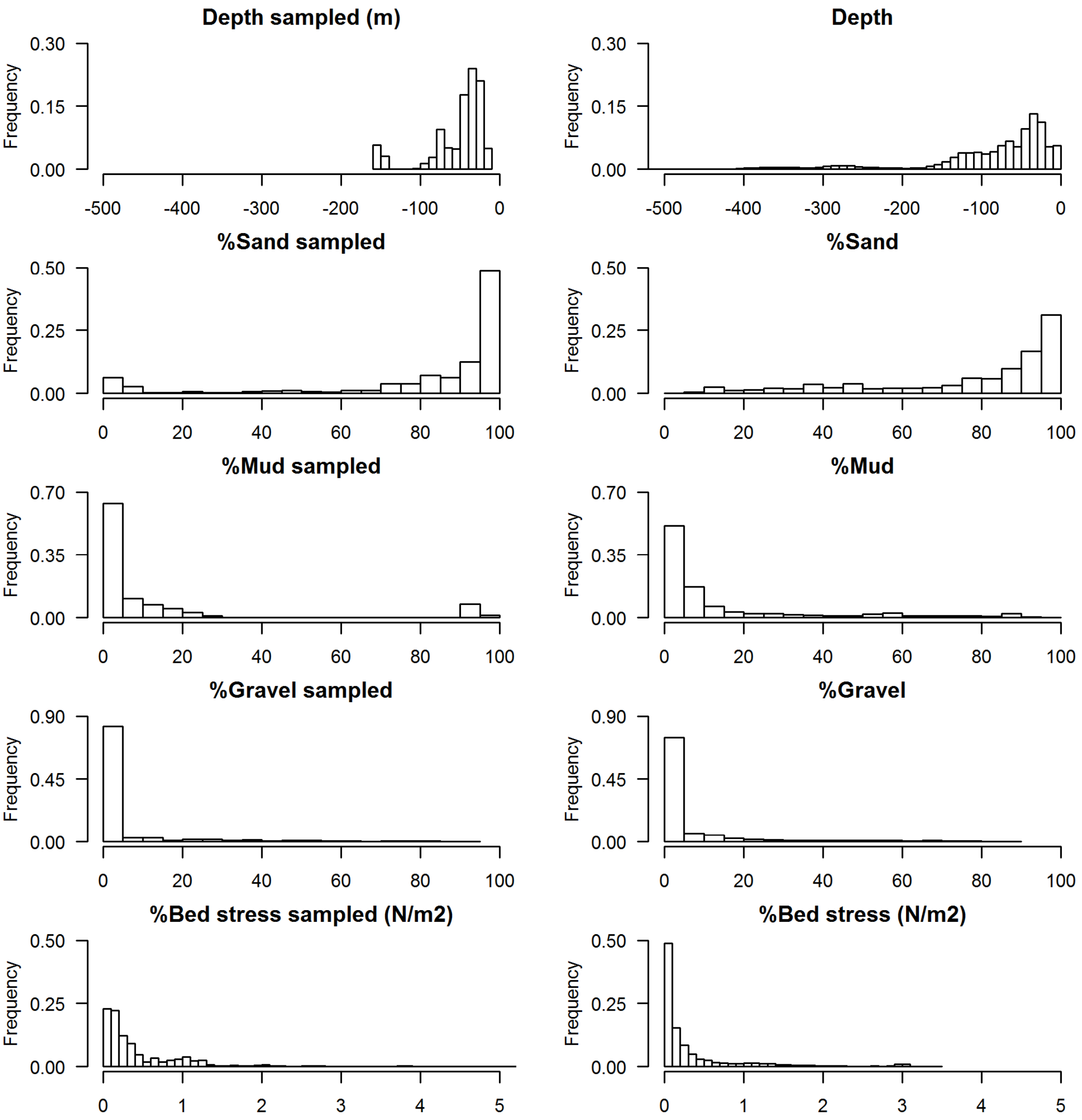

Figure S1. Frequency distribution of environmental variables of the benthos sampling stations (left) and the frequency distribution of environmental variables of all North Sea grid cells (right).
