# Supplementary material for: Estimating sensitivity of seabed habitats to disturbance by bottom trawling based on the longevity of benthic fauna
Source: Ecol Appl. 2018 May 24;28(5):1302–12. doi: 10.1002/eap.1731 (PMC6055734; doi:10.1002/eap.1731)
Supplement: Supplementary file 2 [file EAP-28-1302-s002.pdf]

Appendix S2

Ecological Applications

Estimating sensitivity of seabed habitats to disturbance by bottom trawling based on the longevity of benthic fauna.

Adriaan D. Rijnsdorp[1,2], Stefan G. Bolam[3], Clement Garcia[3], Jan Geert Hiddink[4], Niels T. Hintzen[1], P. Daniel van Denderen[5], Tobias van Kooten[1,6]

[1] Wageningen Marine Research, P.O. Box 68, 1970 AB IJmuiden, The Netherlands;

[2] Aquaculture and Fisheries Group, Wageningen University, P.O. Box 338, 6700 AH Wageningen, The Netherlands;

[3] The Centre for Environment, Fisheries and Aquaculture Science, Pakefield Road, Suffolk NR33 0HT, UK;

[4] School of Ocean Sciences, Bangor University, Menai Bridge, Anglesey, LL59 5AB, UK ;

[5] Centre for Ocean Life, National Institute of Aquatic Resources, Technical University of Denmark, Kemitorvet B-202, 2800 Kongens Lyngby, Denmark;

[6] Institute for Biodiversity and Ecosystem Dynamics, University of Amsterdam, Amsterdam, The Netherlands.

Corresponding author: Adriaan D. Rijnsdorp (adriaan.rijnsdorp@wur.nl)

Critical trawling intensity for a reduction in the relative biomass of long-lived taxa

The critical trawling intensity at which the biomass of long-lived taxa is reduced to a certain proportion (*p*) of the untrawled biomass B_{ref} is derived as follows.

The estimated cumulative biomass – longevity relationship is

$$\ln\frac{B_L}{1-B_L} \sim \beta_0 + \beta_1 \ln(L) + \beta_2 H + \beta_3 \ln(T) + \beta_4 \ln(T) * H + \beta_5 \ln(L) * H + \beta_6 \ln(L) * \ln(T) \quad \text{[S1]}$$

where L is longevity, T is the annual trawling intensity and H is the habitat variable. This equation can be rearranged to obtain an equation for the cumulative biomass proportion B_L of taxa with longevity $\leq L$:

$$B_L = \frac{exp(\beta_0+\beta_1 \ln(L)+\beta_2 H+\beta_3 \ln(T)+\beta_4 \ln(T)*H + \beta_5 \ln(L)*H + \beta_6 \ln(L)*\ln(T))}{1+exp(\beta_0+\beta_1 \ln(L)+\beta_2 H+\beta_3 \ln(T)+\beta_4 \ln(T)*H + \beta_5 \ln(L)*H + \beta_6 \ln(L)*\ln(T))} \quad \text{[S2]}$$

[S1] can also be rearranged to obtain an equation for trawling intensity:

$$\ln(T) = \frac{(ln(B_L/(1-B_L))-(\beta_0+\beta_1 \ln(L)+\beta_2 H + \beta_5 \ln(L)*H)}{\beta_3 + \beta_4 H + \beta_6 \ln(L)} \quad \text{[S3]}$$

The reference biomass B_{ref} = $1 - B_{10}$ (Figure SM2) of the untrawled habitat can be calculated from [S2] by setting longevity to 10 years and trawling intensity to a value of 0.01 yr^{-1}.

$$B_{ref} = 1 - \frac{exp(\beta_0+\beta_1 \ln(10)+\beta_2 H \ +\beta_3 \ln(0.01)+\beta_4 \ln(0.01)*H \ + \beta_5 \ln(10)*H \ + \beta_6 \ln(10)*\ln(0.01))}{1+exp(\beta_0+\beta_1 \ln(10)+\beta_2 H \ +\beta_3 \ln(0.01)+\beta_4 \ln(0.01)*H \ + \beta_5 \ln(10)*H \ + \beta_6 \ln(10)*\ln(0.01))} \quad \text{[S4]}$$

We then define the critical trawling intensity $T_c(p)$ as the trawling intensity at which the biomass proportion of long-lived taxa (L > 10 years, B_{ref}) is reduced to a fraction *p* of its value in absence of trawling. This critical trawling intensity can be obtained by substituting $B_L = 1 - \rho B_{ref}$ in equation [S3]:

$$T_c(p) = exp\left[\frac{ln(1-pB_{ref}/(pB_{ref}))-(\beta_0+\beta_1 \ln(10)+\beta_2 H + \beta_5 \ln(10)*H)}{\beta_3 + \beta_4 H + \beta_6 \ln(10)}\right] \quad \text{[S4]}$$

The above equation includes one habitat variable (*H*), but can be extended to include several habitat variables and their interaction with trawling intensity and longevity.

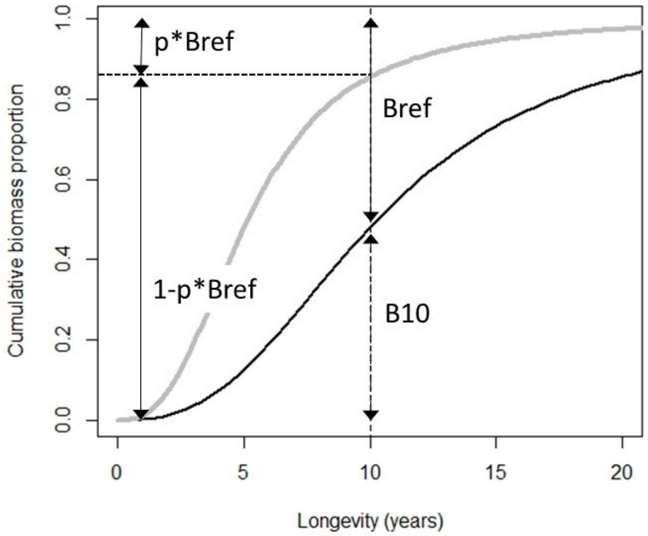

Figure S1. Cumulative biomass distribution of the benthos in an untrawled habitat (black line). The grey line shows the cumulative biomass of the benthos in a habitat where trawling has reduced the biomass of the taxa with a longevity >10 years to $p * B_{ref}$.
